# Gene Expression of Vascular Endothelial Growth Factor A and its Receptors in Dental Pulp of Immature and Mature Teeth

**DOI:** 10.14744/eej.2021.86580

**Published:** 2021-11-19

**Authors:** Jose Francisco GOMEZ-SOSA, Javier CAVIEDES-BUCHELI, Luis Eduardo Diaz BARRERA

**Affiliations:** 1. Department of Endodontics, Universidad Central de Venezuela, Caracas, Venezuela; 2. Centro de Investigaciones Odontológicas, Faculty of Dentistry, Pontificia Universidad Javeriana, Bogotá; 3. Department of Chemical Engineering, Faculty of Engineering, Universidad de la Sabana, Chía, Colombia

**Keywords:** Angiogenesis, dental pulp, NRP1, VEGFA, VEGFR1, VEGFR2

## Abstract

**Objective:**

Vascular endothelial growth factor A (VEGFA) and its receptors are essential proteins for the angiogenic activity of dental pulp. Angiogenesis fundamentally provides oxygen and nutrients to cells for root formation and defence mechanisms. The angiogenic potential of dental pulp should be understood and considered for the conservative and regenerative endodontics. The purpose of this research was to measure the VEGFA expression and its receptors such as vascular endothelial growth factor receptors 1, -2 (VEGFR1, VEGFR2) and Neuropilin 1 (NRP1) in human dental pulp from molars with immature and mature apexes.

**Methods:**

VEGFA system mRNAs expressions were assessed in dental pulp obtained from freshly extracted human third molars divided into immature (n=8) and mature (n=8) apexes. RNAs were extracted from the samples. Each sample's cDNA was synthetized and the target genes VEGFA, VEGFR1, VEGFR2, NRP1 expression profiles obtained by RT2-PCR. Analysis was based on the Student's t-test comparing the replicate 2-ΔCt: values for each gene. P values of <0.05 were considered significant.

**Results:**

In teeth with mature apexes, VEGFA (P=0.0002), NRP1 (P=0.0001), VEGFR1 (P=0.0057) and VEGFR2 (P=0.018259) significantly increased statistically with respect to the immature apexes group.

**Conclusion:**

Within the limitation of the present investigation, it can be concluded that the angiogenic process seems to be a physiological process in the dental pulp due to the studied angiogenic growth factor are expressed in both immature and mature dental pulps. VEGFA and its receptors are expressed significantly higher in mature apex teeth than immature apex teeth.

## Introduction

Angiogenesis is a highly regulated process of blood vessel formation from pre-existing ones that include multiple steps such as: endothelial cell activation and proliferation, endothelial cell migration and invasion, endothelial cell sprouting, basement membrane formation and maturation of neovascularization. These processes are mediated by growth factors of which the most important is the vascular endothelial growth factor A (VEGFA) (1).

**Highlights**

• Angiogenesis is a continual process in the dental pulp.
• Stimuli such as mastication and occlusal trauma could increase angiogenic activity in the dental pulp.
• Gene expression of angiogenic growth factors are- higher in mature dental pulp than in immature.

VEGFA is a crucial regulator of vascular development during embryogenesis (vasculogenesis) and blood-vessel formation in adults (angiogenesis). VEGFs bind in an overlapping pattern to its receptors known as VEGF receptor-1 and -2 (VEGFR1-2) and to other proteins such as Neuropilin 1 (NRP1) (2). VEGFR1 can be considered as a control mechanism inhibiting the angiogenic process. In the absence of VEGFR1, additional VEGFA ligands bind to VEGFR2, which is the first stage of signal transduction to start angiogenesis (3), although as the angiogenic process continues, it has been observed that VEGFR1 intervene in tip cell formation of the sprouting vessel and is also predominantly found in stalk cells (4).

VEGFR2 is the main transducer of VEGFA effects in endothelial cell differentiation, proliferation, migration and tubulogenesis that allow angiogenesis (5). Another VEGFA receptor NRP1, acts as an enhancer of VEGFR2 to bind VEGFA (6). Additionally, NRP1 has been demonstrated as a critical protein in the formation of the vascular tube by endothelial cells during the angiogenesis progress (5). The binding of VEGFA ligands to its VEGFR1, VEGFR2 and NRP1 receptors allows the dental pulp to produce the necessary angiogenesis to transport the oxygen and nutrients necessary for root formation or as a defence mechanism against mastication, occlusal trauma or the aging process (1, 3, 5, 7).

During root formation, the pulp cells from the dental sac produce dentine, which then allows the formation of cementum and periodontal ligament (8, 9). Therefore, it is imperative that the new blood vessels are formed by vasculogenesis at the embryonic stages and later by angiogenesis in teeth with immature apexes, which is possible by the activity by the activity of VEGFA mediated by VEGFR1-2 and NRP1 that allows endothelial cell differentiation, proliferation, migration, and formation of the vascular tube factors (5, 10).

In mature apex teeth, stimuli such as: mastication, occlusal trauma, the aging process or caries, act as chronic aggressors to the dental pulp causing circulatory disturbances and vascular changes, resulting in low oxygen concentration in the tissue. As the stimuli continue, hypoxia will inevitably induce the defensive response of dental pulp which, in turn, produces reparative dentine to calcify the canal. As nutrition and oxygen are needed for such cellular activity, this is achieved through the interaction of VEGFA and its receptors VEGFR1, VEGFR2 and NRP1 changing the endothelial cells that trigger the angiogenic processes (1, 7, 11-14).

During angiogenesis, the endothelial cells are activated by VEGFA, become motile and invasive and protrude cytoplasmic projections (filopodia). The endothelial cells that develop filopodia are called tip cells, these are in charge of guiding the new sprouts toward the guiding signals found in the extracellular environment (4, 15). The endothelial cells that follow the tip cells are stalk cells, which are responsible for forming the lumen of the sprouts through structures similar to filopodia. Tip cells can join with other sprouts to form anastomoses (4, 15). New connections between cells are stabilized when blood flow begins, the basement membrane is established, and when the vessel acquires pericyte coverage (4).

Regardless of the stimulus that induced the imbalance between the supply and consumption of oxygen, cellular and physiological changes in the pulp, either by root formation and development or as a defence mechanism, that triggered the angiogenic response, angiogenesis follows the same path due to the activity of VEGFA and its receptors VEGFA, VEGFR1, VEGFR2, NRP1 (4, 16).

The purpose of this study was to measure the gene expression of the angiogenic growth factor VEGFA and its corresponding receptors VEGFR1, VGFR2, NRP1 in human dental pulp from extracted third molars with immature and mature apexes.

## Materials and Methods

This study was approved by the ethics committee (N°0463- 2013) in accordance with the Declaration of Helsinki. A convenient sample of 16 patient were recruited. All patients taking part in the study were required to sign a certified consent form. Human dental pulp was obtained from a convenient sample of 16 freshly extracted non-carious and unrestored third molars selected from different healthy patients; patients under medication, smokers, or pregnant women were excluded from the study.

The samples were divided into two groups of 8 teeth. The immature apex group samples were taken from patients aged between 13 and 15 years-old, (4 females and 4 males) whose teeth were fully impacted, with an apical orifice diameter greater than 5 mm mesio-distal or bucco-lingual, which were extracted for orthodontic reasons. The mature apex group samples were taken from patients aged between 33 and 50 years-old (4 females and 4 males) whose teeth were in normal occlusion and without periodontal disease, with an apical orifice diameter of not greater than 0.5 mm extracted for causing morsicatio buccarum. Determination of root development was done both radiographically by millimeter periapical radiographs and visually using an operative microscope at 15X magnification by means of an endodontic ruler.

The root surface of each tooth was scraped with a blade to remove the attached periodontal ligament (PDL) that could contaminate the pulp sample. The teeth were then sectioned using a high-speed hand piece with a Zekrya bur (Dentsply Tulsa Dental, OK, USA) irrigated with saline solution. The pulp sample was obtained and processed according to Gomez- Sosa et al. (17) methods.

### RNA extraction

Total RNA was extracted from each sample using a commercially available kit (RNeasy" minikit; Qiagen, Chatsworth, CA, USA). The frozen pulp samples were disrupted using a mortar and pestle and homogenized using a sterile plastic syringe and a 20-gauge (0.9 mm) needle. The lysate was passed through the syringe 10 times or until a homogeneous lysate was obtained in a lysis buffer containing 14.3 M ß-mercaptoethanol. The samples were treated as indicated by the manufacturer and an on- column DNase digestion with an RNase-Free DNase Set (Qiagen) was undertaken. Quality control was performed to ensure that 28S and 18S ribosomal RNA (rRNA) bands were easily identifiable in all of the RNA samples. This involved electrophoresis through a 1.2% agarose gel followed by staining with SYBR Green (Invitrogen, UK) using Leon Marker 30μg 70ng^l (Qbio- gene, USA). The RNA was then quantified using the NanoDrop 2000c spectrophotometer (Thermo Scientific, USA) and Firststrand complementary DNA (cDNA) synthesis was performed using an RT2 First Strand Kit (Qiagen) using 0^g of total RNA as the starting material, according to the manufacturer reverse transcription protocol and Gomez-Sosa et al. (17).

### RT2-PCR

RT2 SYBR Green Mastermix (Qiagen, Chatsworth, CA, USA) was added to each synthetized cDNA sample. 25μ! of that mix was added to RT2 Profiler PCR Arrays (Qiagen, Chatsworth, CA, USA). The RT2 Profiler arrays consisted of custom made 96-wells plates containing seven genes involved in the cell signaling of angiogenic growth factors and their receptors. The target genes were VEGFA, VEGFR1, VEGFR2, NRP1 (Qiagen, Chatsworth, CA, USA, Kit array catalogue number: caph13374) (Table 1). The expression profiles of the target genes were measured relative to the mean critical threshold (CT) values of two housekeeping genes, i.e., glyceraldehyde-3-phosphate dehydrogenase (GAPDH) and actin beta (ACTB). The amplification procedure was checked using three controls: the genomic DNA control, the reverse-transcription control and the positive PCR control according to Gomez-Sosa et al. (17).

**Table 1.** VEGFA and receptors used

Gene ID	GenBank	Gene Name	Description
7422	NM_003376	VEGFA MGC70609, MVCD1	Vascular endothelial growth factor A
3791	NM_002253	VEGFR2, KDR	Vascular endothelial growth factor 2, Kinase insert domain receptor
2321	NM_002019	VEGFR1 FLT1	Vascular endothelial growth factor receptor 1, Fms-related tyrosine kinase 1
8829	NM_003873	NRP1, VEGF165R, CD304	Neuropilin 1

VEGFA: Vascular endothelial growth factor A, VEGFR2: Vascular endothelial growth factor receptor 2, VEGFR1: Vascular endothelial growth factor receptor 1, NRP1: Neuropilin 1

### Sample analysis

Each 96-well plate was placed in a real-time cycler (Light Cycler 96, Roche, Denmark) programed according to manufacturer instructions to perform relative quantification: Pre-incubation 1 cycle, Amplification 45 cycles and Melting 1 cycle, reaction volume 25μl and temperature: Ramp 4.4°C/s Duration: 600 s. Target 95°C. The Ct value of each well plate was calculated by the AACT method using a Microsoft Excel spreadsheet containing algorithms provided by the manufacturer. Quantification was performed in each group to a ΔΔCt of a constitutive expression gene such as GAPDH and ACTB.

### Statistical analysis

P values were calculated based on a Student test comparing the replicate ACt values for each gene. Significance was set at P<0.05. And then the Livak-Schmittgen methods to calculate the relative gene expression of the real time quantitative PCR and the 2(-Delta Delta CT) were performed, using the software provided as a service in the Qiagen web site (Qiagen, Chatsworth, CA, USA).

## Results

In teeth with mature apexes, the expression of VEGFA (P=0.0002), NRP1 (P=0.0001), VEGFR1 (P=0.0057) and VEGFR2 (P=0.0183) significantly increased with respect to the immature apexes group, as shown in Table 2.

**Table 2.** Expression of VEGFA and its receptors in immature vs. mature teeth

Position	Gene	Average ΔCT	2^(ΔΔCT)	P value	Fold up- or Down- Reg
		Immature	Mature	Immature	Mature		Mature/Immature
1	VEGFA	6.41	4.27	0.011739	0.051810	0.0002*	4.41
2	NRP1	3.54	2.20	0.085971	0.217732	0.0001*	2.53
3	VEGFR2	6.49	5.81	0.0111 25	0.017848	0.01826*	1.60
4	VEGFR1	5.19	4.24	0.027323	0.052899	0.0057*	1.94
5	ACTB	-1.10	-1.20	2.141690	2.296402	0.3690	-1.07
6	GAPDH	1.10	1 .20	0.466921	0.435464	0.4262	1.07

VEGFA: Vascular endothelial growth factor A, NPR1: Neuropilin 1, VEGFR2: Vascular endothelial growth factor receptor 2, VEGFR1: Vascular endothelial growth factor receptor 1, CT: Threshold cycle, ACTB: Actin beta, GAPDH: Glyceraldehyde 3-phosphate dehydrogenase. *Results with significant difference

## Discussion

VEGFA and its receptors VEGFR1, VEGFR2 and NRP1 were chosen because they manifest almost all of the angiogenic activity in dental pulp. These growth factors under hypoxia, caused by the increased cellular activity necessary for the production of hard tissue, either for the formation of roots in immature teeth or as a defence mechanism in mature teeth, will be expressed to produce new blood vessels that restore the normoxia necessary for the cellular activity in the pulp (1, 6, 18).

VEGFA had a greater expression in mature apex pulps (P=0.000241), where the apex is further closed, and there is greater angiogenic activity. This is due to the intense cellular activity providing defence mechanisms, for the generation of hard tissue against stimuli such as mastication, occlusal trauma, bruxism and tissue aging (1, 10-12, 17-20) which coincides with a study carried out in patients subjected to orthodontic forces and occlusal trauma, showing that VEGF was significantly higher in teeth subjected to those forces, demonstrating that stimuli are additive on the dental pulp tissue been effective triggering the angiogenic activity (21). In immature teeth pulp, VEGFA is also expressed for maintaining the oxygen concentration levels and nutrients for root formation (1, 17).

VEGFA receptors vary according to the endothelial cell's biological requirements. In this research VEGFR1 had a higher expression in mature apex pulp (P=0.0057). This is explained as an angiogenic control mechanism. VEGFR1 competes with VEGFR2 to bind to VEGFA (3) only allowing sufficient vessels to be formed, since it was expressed in stalk cells and also intervenes in tip cell formation of sprouting vessels, regulating the oxygen concentrations necessary for cellular function in the formation of hard tissue for both root defence mechanisms in mature teeth, and root development in immature teeth (4).

VEGFR2 showed a higher expression in mature apex pulps (P=0.0183), due to the active sprouting of vessels. This demonstrates that dental pulp cells are in a state of hypoxia due to increased cell formation, as a defence mechanism, as new blood vessels are required to provide additional oxygen for cellular activity (1, 10-12, 19, 20).

The primary function of NRP1 is to enhance VEGFA signaling via the VEGFR2 receptor and act on endothelial cells for vascular tube formation (5, 6, 22). In this research NRP1 showed a statistically significant difference, (P=0.0001), in mature apex teeth. Where there is a greater requirement for new blood vessels to carry the oxygen and nutrients for the formation of hard tissue as a defence mechanism (5). Conversely, activated NRP1 can lead to endothelial destabilization and paracellular permeability independently of VEGFR2, and behave as an independent receptor (5, 23). From the results of this research, it can be inferred that in dental pulp NRP1 does behave like an independent receptor, so that angiogenesis can be initiated following the VEGFA/NRP1 pathway (5, 23).

From the results of this biomolecular research, several clinical implications can be inferred: In mature apex teeth, VEGFA, VEGFR1-2 and NRP1 have a statistically significant difference in their gene expression, indicating intense angiogenic activity, with the maturest tooth having the greatest angiogenic activity. This angiogenic activity is required for maintaining sufficient oxygen levels for cellular and functional change as a defence mechanism against noxious stimuli (1, 10-12, 19, 20).

In immature apex dental pulp, it was observed that VEGFA, VEGFR1-2 and NRP1 genes statistically significantly decrease their expression, indicating that angiogenic activity is less than that in mature teeth. This can be explained in different ways: the existing vasculature is sufficient to provide oxygen to the cellular components involved in root formation, or cells proliferate significantly more in hypoxia than in normoxia (24).

Similar results were found in equal dental groups with the same root characteristics: immature teeth with apical foramen greater than 5mm (16-19 years) and mature teeth with apical foramen not greater than 0.5mm (17-29 years). In which it was observed that HIF-1a, ANG1, ANG2 and TIE2 were significantly higher in mature teeth than in mature ones, showing that other proteins with angiogenic potential follow the same trend as the present investigation (25).

This data shows us that angiogenesis is a continual process, growth factors with angiogenic potential are expressed in both immature and mature dental pulp at different stages of apical maturation. What varies is the magnitude of its expression, coinciding with the observations of Güven et al. (26) who found a moderate expression of VEGF in the pulp of third molars. Additionally, the research of Grando et al. (27) also observed in young permanent teeth, by immunohistochem- istry, a uniform layer in some endothelial cells indicating the presence of VEGFR2. Moreover, the findings of Virtej et al. (28) showed that all proteins members of the VEGF family (A, B, C and D) are expressed in dental pulp and also found VEGFR2 and VEGFR3 receptors in vascular and immunological cells of the dental pulp. These authors suggest that the expression of these proteins would be indicative of pulpal physiological angiogenesis, and corroborate the findings of this present study.

## Conclusion

The angiogenic growth factor VEGFA and its receptors VEGFR1-2 and NRP1 genes are expressed in both mature and immature apex dental pulp as measured by RT2-PCR, but they significantly increase in mature apex teeth.

### Disclosures

**Acknowledgements:** The authors would like to thank Yepsel Rada, Birmania Duque, Daniel Rada and Ian Garret for their comments and support.

**Conflict of interest:** The authors deny any conflict of interest.

**Ethics Committee Approval:** This study was approved by the ethics committee of the Faculty of Dentistry of the Universidad Central de Venezuela (Date: 07/25/2013, Number: N 0463-2013).

**Peer-review:** Externally peer-reviewed.

**Financial Disclosure:** This study did not receive any financial support.

**Authorship contributions:** Concept - J.F.G.S., J.C.B.; Design - J.F.G.S., J.C.B., L.E.D.B.; Supervision - J.F.G.S.; Funding - J.F.G.S.; Materials - J.F.G.S.; Data collection &/or processing - J.F.G.S., L.E.D.B.; Analysis and/or interpretation - J.F.G.S., L.E.D.B.; Literature search - J.F.G.S., J.C.B.; Writing - J.F.G.S., J.C.B.; Critical Review - J.F.G.S., J.C.B.
